# Myocardial Infarction in a 7-Year-Old Girl with Polyarteritis Nodosa

**DOI:** 10.1155/2022/2175676

**Published:** 2022-04-26

**Authors:** Lina Bayazeed, Alaa Felimban, Abdulsalam Alsaiad, Fahd Alsufiani, Jubran Alqanatish

**Affiliations:** ^1^King Saud Bin Abdulaziz University for Health Sciences, Riyadh 14611, Saudi Arabia; ^2^King Abdullah International Medical Research Center (KAIMRC), Riyadh 14611, Saudi Arabia; ^3^King Abdullah Specialist Children's Hospital, Riyadh 14611, Saudi Arabia; ^4^King Abdulaziz Medical City (National Guard Health Affairs), Riyadh 14611, Saudi Arabia

## Abstract

Polyarteritis nodosa (PAN) is a rare systemic vasculitis that affects small to medium-sized arteries. It could affect any organ including the heart. However coronary artery involvements are very rare. We describe a young girl who presented following a histopathological diagnosis of PAN with acute chest pain, high serum troponin, and progressive ischemic changes in the electrocardiogram (ECG). Induction of remission of her disease was done with six-moths Cyclophosphamide infusions and pulse corticosteroids. In addition to anticoagulation and dual antithrombotic therapy, the disease remission was maintained with mycophenolate mofetil which helps in the recovery of coronary disease. Our case illustrates the serious cardiac involvement of PAN in a child that responded to intensive management.

## 1. Introduction

Polyarteritis nodosa (PAN) is a necrotizing vasculitis that affects the wall of small to medium-sized arteries. The clinical spectrum is highly variable where it can be systemic or localized to one organ or system [1]. PAN occurs primarily in middle-aged adults but appears even more rarely in children where both girls and boys are affected equally [2]. It occurs in different forms as cutaneous (the most common form), classic, systemic, or microscopic. Cutaneous PAN is necrotizing vasculitis confined to the skin. It is relatively a benign form that doesn't have deep organ involvement in contrast to systemic PAN which is a potentially life-threatening and involve multiple systems like kidney, heart, lung, gastrointestinal tract, liver, nervous and musculoskeletal systems. Microscopic is localized PAN lesions discovered in surgical specimens, such as the appendix, gallbladder, testes, and other internal organs [3]. Surgical excision is usually curative in this form of PAN [1].

The term classic is used to differentiate idiopathic systemic PAN from a group of patients who have a loss of function mutation of the gene encoding adenosine deaminase 2 (ADA2) [4].

In children's skin, MSK, and GIT are frequently involved while cardiac, brain, and lung are less involved [5, 6]. Cardiac involvement in pediatric PAN is much less compared to adults affected with PAN [7].

We report a pediatric patient diagnosed as a medium-sized vasculitis confirmed based on clinical features, histopathology, and elevated inflammatory markers to have PAN; who presented initially as acute abdominal pain underwent a laparoscopic appendectomy and complicated by acute myocardial infarction (AMI) secondary to Coronary artery vasculitis a few days later.

## 2. Case Presentation

A previously healthy, 7-year-old girl presented with a history of persistent abdominal pain and vomiting for 4 days. It preceded by upper respiratory tract infection symptoms the week before patient's presentation. She was admitted for the first time in March 2019, as a case of gastritis and to rule out urinary tract infection (UTI). A previous history of intermittent mild abdominal pain since the age of 2 years was reported. Infrequently, associated with vomiting and diarrhea, which occur 1-2 times per year. No history of fever, weight loss, skin rash, respiratory symptoms, headache, or urine changes. Family history was unremarkable for similar presentation, genetic disorders or vasculitis.

**Figure 1:**
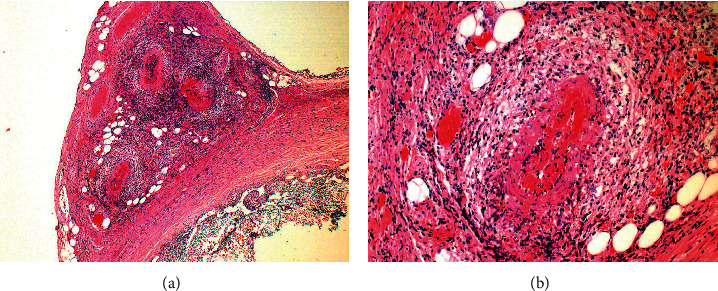
(a) Mesoappendix and part of the appendix. Necrotizing vasculitis in mesoappendix small- to medium-sized arteries is evident. There is no inflammation in the appendix mucosa and muscularis propria. Low magnification. H & E stained slide. (b) Segmental transmural necrotizing inflammation. In the involved artery/arteriole, there is fibrinoid necrosis, neutrophils and eosinophils which associated with lumen narrowing. There are no granulomas. High magnification. H & E stained slide.

Laboratory investigations showed high White cell counts, Erythrocyte Sedimentation Rate, and C-Reactive Protein (WBC 14.6 × 109/L, ESR 70 mm/hr, CRP 179 mg/L). Due to her severe abdominal pain, she underwent an abdominal ultrasound, which was suspicious for appendicitis; the appendix measured 0.65 cm with mild free fluid in the right lower quadrant. The patient was taken to the operating room for laparoscopic appendectomy. Intra-operative the surgeon noticed that the appendix looked non-inflamed so it was sent for histopathological examination. The histopathology report showed; Focal peri-appendicular tissue with 3 medium-sized vessels showing necrotizing vasculitis, there were also few small size vessels with the same finding and no evidence of appendicular inflammation (Figures 1(a) and 1(b)).

The rheumatology team was consulted and the diagnosis of polyarteritis nodosa was made, based on clinical features, laboratory, and histopathology findings. She received three doses of intravenous (I.V) pulse steroid. During hospitalization, an echocardiogram was requested for coronary arteries evaluation and showed normal intra-cardiac anatomy and function with normal coronary arteries' size and branching. No ischemic changes were found in the electrocardiogram. Ophthalmology evaluation showed a normal ocular exam for both eyes. Urinalysis and renal Doppler ultrasound came normal. CT Angiogram of the abdominal vessels (Hepatic, renal, celiac, splenic, mesenteric arteries) showed no findings of vasculitis. Antineutrophil cytoplasmic antibodies (cANCA and pANCA) were negative. Cyclophosphamide was planned to be given in Day Care Treatments Unit (DCTU) 10 days after discharge to allow proper surgical wound healing. The patient was discharged home on prednisolone 2 mg/kg/day divided doses and to be followed in the Rheumatology clinic within a week.

**Figure 2:**
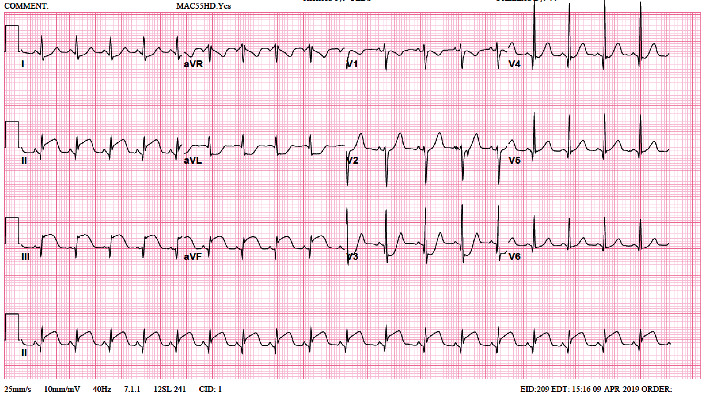
Ischemic changes involving right coronary territories.

**Figure 3:**
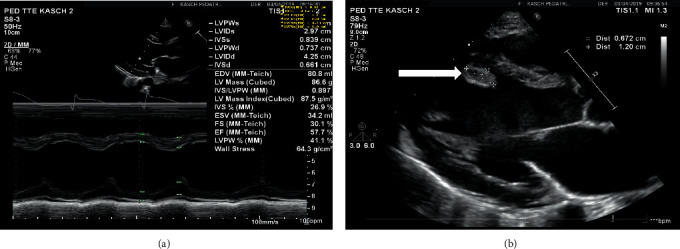
(a) Impaired left ventricular systolic function. (b) Left ventricular thrombus (white arrow).

On the 3rd day after discharge, the patient presented to the ER with a complaint of persistent left-sided chest pain, moderate to severe, localized, not radiating. No abdominal pain, fever or other symptoms. Laboratory workup showed a remarkably high Troponin I level of 1682 pg/mL. Initially, ECG and Echocardiogram were normal. The patient was admitted to the Pediatric Intensive Care (PICU) for close monitoring. Nine hours later on the same day, cardiac enzymes kept in trending up (Table 1) and ischemic changes start to appear in the electrocardiogram in form of ST elevation in inferior leads and ST depression in lead I and AVL which is consistent with RCA territory (Figure 2). Urgently repeated echocardiogram showed; impaired left ventricular systolic function involving the septal wall and anterior LV wall; Left anterior descending artery territories (Figure 3(a)). A serial echocardiogram was repeated a few hours later showed multiple echogenic masses at the left ventricular apex (1.5 × 1 cm), LV inferior wall, and in the right ventricle most likely thrombus (Figure 3(b)). The patient's chest pain was progressive and excoriating, that required morphine infusion. Also, she started to have elevated systolic blood pressure, which was controlled by amlodipine and as needed hydralazine. Nitroglycerin and heparin infusion was initiated and she was transferred immediately to the Pediatric Cardiac Intensive Care Unit (PCICU).

**Figure 4:**
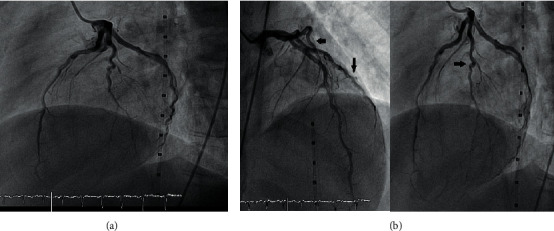
(a) None obstructed coronary arteries. (b) Irregularity of coronary arteries (black arrows).

The multidisciplinary meeting(MD) was conducted including cardiology, rheumatology, intensive care, and hematology teams, and the patient was taken to the cardiac catheterization lab for a coronary angiogram and possible thrombus removal. Coronary angiography showed no coronary obstruction in both left and right coronary artery systems (Figure 4(a)) but it showed irregularity of the left main coronary artery which indicating vasculitis (Figure 4(b)).

**Figure 5:**
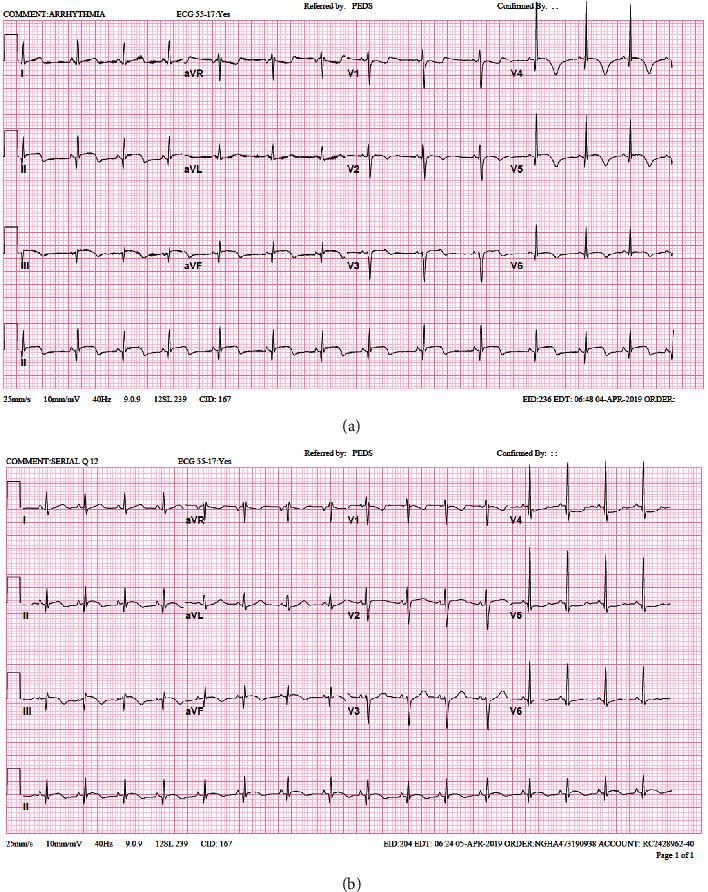
(a) ECG with ischemic changes. (b) Normal ECG on the 4^th^ day after treatment.

The MD decided to add Aspirin, Clopidogrel, and to give one dose of IVIG 2 g/kg and start intravenous methylprednisolone (2 mg/kg). 2 days later, all cultures came negative and thrombophilia workup was unremarkable. However, the patient was still in critical condition and requiring inotropic and ventilator support so one dose of Infliximab 6 mg/kg/dose was given. ECG at that time is showing ischemic changes in form of ST elevation in inferior leads and ST depression in lead I and AVL (Figure 5(a)).

Follow-up ECG on the 4th day of admission showed no more ischemic changes (Figure 5(b)) and repeated echocardiogram showed improved cardiac function with regression in the size of the left ventricle clot. Further laboratory test results revealed high von willebrand factor >199.5%, negative ANA, normal complements, and immunoglobulins levels. Renal and liver functions remain normal. Whole exome sequencing (WES) was sent to exclude adenosine deaminase 2 deficiency and was negative for CECR1 mutations.

Cyclophosphamide 500 mg/m^2^ was initiated as previously planned and the Tropinin I level continues to improve (Table 1). The patient was weaned off inotropes and ventilator over two weeks and was discharged on the third week from hospital in a stable condition with close follow-up.

The patient completed the induction phase of 6 doses of monthly I.V Cyclophosphamide 500 mg/m^2^. Then, she was maintained on oral mycophenolate mofetil 600 mg/m^2^/day every 12 hrs. Heparin and anti-thrombotic agents were continued. Corticosteroid was weaned off slowly over 10 months with follow up of cardiac enzymes, inflammatory markers, and cardiac function.

**Figure 6:**
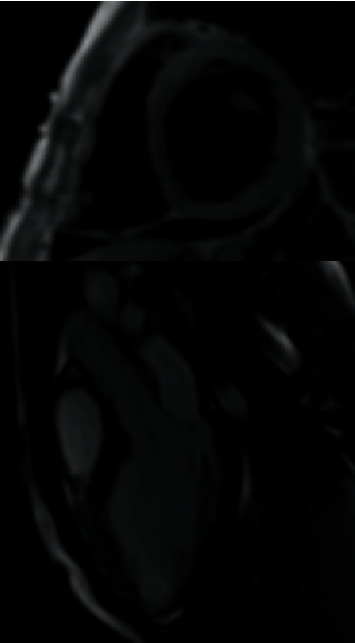
Cardiac MRI showed mildly dilated LV.

Cardiac MRI was done 3 months after discharge which showed mildly dilated LV with normal systolic function 69%. There are LV wall motion abnormalities (hypokinesia), abnormal thinning of the apical septal and lateral wall, and diffuse LV mid-wall scarring involving most of the LV mid & apical segments as well as the apical cap with normal coronary arteries (Figure 6).

The patient completed 24 months of follow-up on ASA, Clopidogrel, and mycophenolate mofetil with no signs of inflammation and normal cardiac function.

## 3. Discussion

In children, Ozen et al. reported gastrointestinal symptoms in 24% of 63 children with PAN [6]. Another study evaluated the cardiac findings of 15 children with childhood PAN [2]. The most common findings were diminished left ventricular systolic function and mild mitral and/or tricuspid valve regurgitation. No cases were reported to have myocardial infarction [2].

In a single center experience over thirty two years, among 69 children diagnosed with PAN, cardiac involvement was present in 3 patients (4%), with 2 of them had valvular heart disease and 1 had pericarditis. Another 3 patients were found to have silent involvement of the coronary arteries during screening echocardiography [5].

In the combined pediatric and adult cohort of polyarteritis nodosa patients reported by Erden et al., gastrointestinal and cardiovascular involvement were more common in adults than children; 31.5% versus 15.5% and 19% versus 4.4% respectively, (*P* value < 0.001) [5–8]. In a patient with PAN abdominal pain is considered the most common presenting symptom of gastrointestinal involvement [5]. There are multiple cases in the literature reported as PAN presented with acute abdominal pain that mimics appendicitis [9]. However, necrotizing vasculitis of the appendix associated with PAN is uncommon; the largest series in the literature consists of 12 adult cases reported a close relationship between necrotizing arteritis of the appendix and PAN [10]. Similarly, our case presented with acute appendicitis and necrotizing vasculitis.

In clinical and autopsy of five series including 283 adult patients with PAN, congestive heart failure was found to be the most common cardiac involvement (27%), while coronary involvement with myocardial infarction was rare (2%) [11, 12].

Coronary involvement with secondary myocardial infarction in PAN was rarely reported in clinical practice for adults and children, and most of the reported cases were found in postmortem studies [5, 6].

In the largest cohort of 69 children mentioned above, all patients received induction therapy with a combination of corticosteroids and cyclophosphamide, then maintained on either azathioprine or mycophenolate with low-dose corticosteroids. All had achieved remission, with a median of 2 months (range 1–24 months) from diagnosis to remission [5]. The study didn't include specific details about each case; however, it didn't report any relapse, morbidity, or mortality among these cases.

The 5-year survival of patients with PAN without treatment is 13%, while with optimal therapy, it increases to approximately 80% [7]. Early diagnosis and early aggressive treatment are essential and can prevent long-term morbidity and mortality [7].

In the current study, we report a pediatric case of polyarteritis nodosa who presented initially with GI manifestation, treated by 3 doses of pulse methylprednisolone and then complicated after few days by coronary arteritis with secondary myocardial infarction that leads to depressed LV function with small intraventricular thrombus. The patient responded well to systemic steroid, Infliximab, and Cyclophosphamide. In addition to anticoagulation and anti-thrombotic therapy. Her remission was maintained by mycophenolate mofetil and satisfactory cardiac outcome was achieved on follow up.

Polyarteritis nodosa in pediatric is a rare but serious condition. It is clear that early and aggressive multidisciplinary treatment plan leads to favorable outcome.

## Figures and Tables

**Table 1:** Troponin I trend in the patient over first week of admission.

Exam name	Reference value	1^st^ day of admission	2^nd^ day of admission	3^rd^ day of admission	4^th^ day of admission	5^th^ day of admission
Troponin I	<15.5 pg/mL	1682 then repeated 6019	19835	50.000	42529	29057

## Data Availability

None.
